# Omega-3 Fatty Acids Attenuate LPS-Induced Acute Kidney Injury via Activation of AMPK/SIRT1/PGC-1α/NRF2/FOXO3 Signaling and Suppression of NF-κB-Mediated Inflammation

**DOI:** 10.3390/nu18040618

**Published:** 2026-02-13

**Authors:** Bindal Ahmet, Asci Halil, Kolay Oznur, Tepebasi Muhammet Yusuf, Kizilkaya Sinan, Ilhan Ilter, Bindal Tozlu Gulsum, Ozmen Ozlem

**Affiliations:** 1 Department of Anesthesiology and Reanimation, Faculty of Medicine, Suleyman Demirel University, Isparta 32260, Türkiye; sinankizilkaya@sdu.edu.tr; 2 Department of Pharmacology, Faculty of Medicine, Suleyman Demirel University, Isparta 32260, Türkiye; halilasci@sdu.edu.tr; 3 Department of Pharmacology, Institute of Health Sciences, Suleyman Demirel University, Isparta 32260, Türkiye; oznurk753@gmail.com; 4 Department of Medical Genetic, Faculty of Medicine, Suleyman Demirel University, Isparta 32260, Türkiye; muhammettepebasi@sdu.edu.tr; 5 Department of Biochemistry, Faculty of Medicine, Suleyman Demirel University, Isparta 32260, Türkiye; ilterilhan@sdu.edu.tr; 6 Burn Unit, Isparta City Hospital General Surgery Clinic, Isparta 32200, Türkiye; drtozlu333@yahoo.com; 7 Department of Pathology, Faculty of Veterinary Medicine, Burdur Mehmet Akif Ersoy University, Burdur 15030, Türkiye; ozlemoz@mehmetakif.edu.tr

**Keywords:** HSP70, Interleukin 6, oxidative stress, apoptosis, Heme oxygenase 1, omega-3 fatty acids, inflammation

## Abstract

**Background:** Sepsis-induced acute kidney injury (AKI) involves oxidative stress and inflammation. Omega-3 (OMG-3) fatty acids possess antioxidant properties, but their impact on the mitochondrial AMPK/SIRT1/PGC-1α/NRF2/FOXO3 axis in AKI remains unclear. **Methods:** Thirty-two male Wistar rats were divided into Control, LPS (5 mg/kg), LPS + OMG-3 (500 mg/kg/day), and OMG-3 groups. Renal tissues were analyzed for histopathology, immunohistochemistry (*TNF-α*, *Caspase-3*, *HSP70*), and gene expression (*AMPK*, *SIRT1*, *PPGC-1α NRF2*, *FOXO3*). **Results:** LPS caused severe tubular injury, increased *TNF-α*, *Caspase-3*, and *HSP70* expression, while significantly downregulating the AMPK/SIRT1/PGPGC-1αRF2/FOXO3 signaling pathway. OMG-3 treatment alleviated histopathological damage, suppressed inflammatory and apoptotic markers, and restored the expression of mitochondrial and antioxidant genes. **Conclusions:** OMG-3 fatty acids protect against LPS-induced AKI by upregulating the gene expression of components in the *AMPK/SIRT1/PGCPGC-1αF2/FOXO3* axis and suppressing NF-κB-driven inflammation. This dual regulation highlights OMG-3 as a potential therapeutic agent for sepsis-related renal injury.

## 1. Introduction

Acute kidney injury (AKI) is a severe clinical syndrome characterized by the sudden loss of renal function due to ischemic, toxic, or inflammatory insults [1]. Among experimental models, Lipopolysaccharide (LPS)-induced sepsis has been widely employed to mimic systemic inflammation and oxidative stress-mediated renal dysfunction [2]. LPS, a major component of the Gram-negative bacterial cell wall, binds to Toll-like receptor 4 (TLR4) on renal tubular and endothelial cells, activating Nuclear factor kappa beta (NF-κB)-dependent transcription and leading to overproduction of Tumor necrosis factor alpha (TNF-α), Interleukin 1 beta (IL-1β), and Interleukin 6 (IL-6) [2]. This cytokine surge initiates tubular apoptosis, mitochondrial swelling, and microvascular congestion, ultimately contributing to glomerular atrophy, tubular epithelial degeneration, interstitial infiltration, and collagen accumulation hallmarks of sepsis-associated AKI [3,4].

Mitochondrial dysfunction and redox imbalance are now recognized as pivotal mechanisms in the pathophysiology of AKI [5]. Excessive Reactive oxygen species (ROS) generation disrupts mitochondrial biogenesis and electron transport, leading to lipid peroxidation, energy failure, and cell death [5]. Within this context, the Adenosine monophosphate-activated protein kinase (AMPK)/Sirtuin 1 (SIRT1)/Peroxisome proliferator-activated receptor-γ coactivator 1 alpha (PGC-1α) axis serves as a master regulatory pathway governing mitochondrial function, energy metabolism, and oxidative stress adaptation [6]. Activation of AMPK increases intracellular NAD^+^ (nicotinamide adenine dinucleotide oxidized form) levels, thereby enhancing the deacetylase activity of SIRT1, which in turn deacetylates and stimulates PPGC-1αto promote mitochondrial biogenesis and antioxidant gene transcription [7]. Downstream, NRF2 and FOXO3—multifunctional transcription factors regulating apoptosis, autophagy, cell cycle arrest, and antioxidant defense—coordinate cytoprotective and proteostatic responses to preserve redox balance under inflammatory stress [8]. Although LPS exposure may trigger an initial adaptive stress response involving heat shock protein 70 (HSP70), sustained endotoxemia primarily drives NF-κB activation while suppressing or overwhelming the NRF2-mediated antioxidant defense [9]. This disruption leads to the inhibition of the AMPK/SIRT1/PGPGC-1αxis, resulting in mitochondrial dysfunction and tubular cell loss [10,11].

Omega-3 (OMG-3) polyunsaturated fatty acids, notably eicosapentaenoic acid (EPA) and docosahexaenoic acid (DHA), exert robust anti-inflammatory, antioxidant, and mitochondrial-protective effects by modulating cellular stress and energy-sensing pathways [12]. OMG-3 supplementation attenuates NF-κB activation, reduces *TNF-α* and *Caspase 3*

(Cas-3) expression, and enhances SIRT1-PGC-1α signaling, thereby restoring mitochondrial function and reducing apoptosis in several organ injury models [13,14]. Moreover, OMG-3 activates NRF2, FOXO3, and HSP70-mediated antioxidant and cytoprotective responses, ultimately improving mitochondrial bioenergetics, protein folding stability, and membrane integrity [15,16]. In renal models, OMG-3 fatty acids reduce tubular necrosis, glomerular congestion, and interstitial inflammation by suppressing oxidative stress markers and promoting mitochondrial recovery [17,18].

Given this background, the present study was designed to elucidate the renoprotective effects of OMG-3 fatty acids in an LPS-induced AKI model by integrating histopathological, immunohistochemical, and molecular approaches. Specifically, we aimed to determine whether OMG-3 modulates AMPK/SIRT1/PGC-1α/FOXO3/HSP70/NRF2 signaling to counteract LPS-induced oxidative, apoptotic, and inflammatory responses in renal tissue. We hypothesized that OMG-3 treatment would restore mitochondrial homeostasis, suppress pro-inflammatory cytokine expression, and mitigate histopathological alterations such as glomerular atrophy, tubular epithelial degeneration, and collagen deposition, thereby demonstrating a mechanistic link between metabolic regulation and renal protection.

Despite extensive work on OMG-3 and inflammation, no study to date has comprehensively examined how OMG-3 regulates the full mitochondrial biogenesis and redox-defense axis (AMPK/SIRT1/PGC-1α → NRF2/FOXO3) in conjunction with apoptotic (Cas-3) and stress-response (HSP70) pathways in LPS-induced AKI. This gap limits the mechanistic understanding of how metabolic reprogramming influences sepsis-associated renal injury. Therefore, this study integrates histopathology, immunohistochemistry, and gene expression profiling to elucidate the multi-layered protective mechanism of OMG-3.

**Figure 1:**
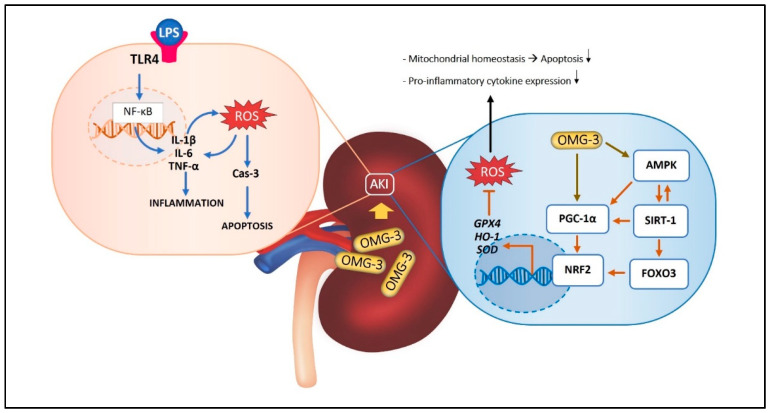
Schematic representation of the protective mechanism of OMG-3 against LPS–AKI. In the left panel, LPS binding to TLR4 activates NF-κB and elevates IL-1β, IL-6, TNF-α, and ROS, triggering Cas-3-mediated apoptosis and inflammation. In the right panel, OMG-3 stimulates the AMPK/SIRT-1/PGC-1α axis, activating NRF2 and FOXO3 to upregulate HO-1, GPX4, and SOD. This cascade restores mitochondrial balance, reduces oxidative stress, and protects renal tissue from LPS-induced AKI. LPS: Lipopolysaccharide, TLR4: Toll-like receptor 4, NF-κB: Nuclear factor kappa beta, IL-1β: Interleukin 1 beta, IL-6: Interleukin 6, TNF-α: Tumor necrosis factor alpha, ROS: Reactive oxygen species, Cas-3: Caspase 3, OMG-3: Omega-3, AMPK: AMP-activated protein kinase, SIRT-1: Sirtuin 1, PPGC-1α Peroxisome proliferator-activated receptor-γ coactivator 1 alpha, NRF2: Nuclear factor erythroid-2-related factor 2, FOXO3: Forkhead box O3, HO-1: Heme oxygenase-1, GPX4: Glutathione peroxidase 4, SOD: Superoxide dismutase, AKI: Acute kidney injury.

The potential effects of OMG-3 on LPS-induced renal injury through the AMPK/SIRT1/PGC-1α/FOXO3/NRF2 signaling pathway are summarized in Figure 1.

## 2. Materials and Methods

### 2.1. Experimental Animals and Ethical Approval

Thirty-two adult male Wistar albino rats (8–10 weeks old, 250–300 g) were used in this study. Animals were obtained from the Suleyman Demirel University Experimental Animal Research Center and acclimatized for one week prior to the experiments under controlled conditions (22 ± 2 °C, 55 ± 5% humidity, 12 h light/dark cycle). A standard pellet diet and water were provided ad libitum. All animal procedures were conducted in accordance with the National Institutes of Health (NIH) Guide for the Care and Use of Laboratory Animals and were approved by the Süleyman Demirel University Local Ethics Committee (Approval No: 10/653; Date: 16 October 2025).

### 2.2. Experimental Design and Treatment Protocol

Rats were randomly divided into four experimental groups, each consisting of eight animals:

1. Control group: Rats received 0.9% saline solution orally for three consecutive days and an intraperitoneal (i.p.) injection of saline on the third day.
2. LPS group: 0.9% saline was administered orally for three days and a single i.p. injection of LPS (Escherichia coli O55:B5; Sigma-Aldrich, St. Louis, MO, USA) on day three to induce AKI [19].
3. LPS + OMG-3 group: Rats received a single LPS injection (5 mg/kg, i.p.) followed by OMG-3 fatty acid (Omegaven^®^, Fresenius Kabi Türkiye, Istanbul, Türkiye) supplementation at a dose of 500 mg/kg/day via oral gavage for three consecutive days [20].
4. OMG-3 group: Rats received only OMG-3 fatty acids for three days to evaluate the compound’s basal effects and an ip injection of saline on the third day.

The dose (500 mg/kg/day) and duration were selected based on established protocols in the previous literature demonstrating robust systemic anti-inflammatory efficacy without significant metabolic toxicity in rats [21]. A specific dose–response study was not performed in the present investigation; instead, a previously validated effective dose was utilized to specifically isolate the mechanistic impact of OMG-3 on the AMPK/SIRT1 axis. At 6 h after LPS injection, animals were anesthetized using ketamine (90 mg/kg, Keta-Control, Doğa İlaç, Türkiye) and xylazine (10 mg/kg, Xylazinbio, Bioveta, Czech Republic). Blood and both kidneys were harvested, and the animals were sacrificed by surgical exsanguination under deep anesthesia.

### 2.3. Reagents and Preparation

LPS was freshly dissolved in sterile saline. The OMG-3 source was the commercially available lipid emulsion Omegaven^®^ (Fresenius Kabi), typically designed for intravenous use. For this study, the emulsion was suspended in corn oil immediately before administration to facilitate oral gavage. All reagents were of analytical grade.

### 2.4. Tissue Collection and Processing

The right kidney was fixed in 10% neutral buffered formalin for histopathological and immunohistochemical analyses, while the left kidney was snap-frozen in liquid nitrogen and stored at –80 °C for biochemical and gene-expression analyses. For biochemical assays, renal tissues were homogenized in ice-cold phosphate-buffered saline (PBS, pH 7.4) using a Teflon glass homogenizer (Ultra-Turrax, IKA, Staufen, Germany). The homogenates were centrifuged at 5000 rpm for 15 min at 4 °C, and the supernatants were collected for further analyses.

### 2.5. Histopathological Evaluation

Kidney tissue samples were fixed in 10% neutral-buffered formaldehyde solution for 48 h and processed by routine histological procedures. The tissues were embedded in paraffin, and 5 µm sections were cut using a rotary microtome. Sections were stained with hematoxylin and eosin for general histological evaluation of the renal architecture.

All slides were examined and photographed using a Nikon Eclipse E-600 photomicroscope (Nikon, Tokyo, Japan) equipped with a NIS-Elements image analysis system. To ensure objectivity, histopathological scoring was performed by a pathologist who was blinded to the experimental treatment groups. Histopathological assessment was based on the presence and severity of the following renal lesions: atrophic glomeruli and renal corpuscles, collagenous material accumulation, tubular dilatation, and tubular epithelial degeneration. Each parameter was semi-quantitatively scored on a scale of 0–3, as previously described by Candan et al. [3].

### 2.6. Immunohistochemical Analysis

For immunohistochemical evaluation, 5 µm sections obtained from paraffin blocks were mounted on poly-L-lysine-coated slides and incubated overnight at 45 °C. The sections were deparaffinized in xylene and rehydrated through a graded ethanol series. Antigen retrieval was performed by heating the sections in citrate buffer (pH 6.0) in a microwave oven (2 × 5 min). Endogenous peroxidase activity was blocked using 3% hydrogen peroxide (H_2_O_2_) for 20 min.

Non-specific binding sites were blocked with Ultra V Block (Thermo Fisher Scientific, Waltham, MA, USA). Sections were then incubated with the following primary antibodies for 60 min at room temperature: TNF-α (Santa Cruz Biotechnology, Dallas, TX, USA sc-52746; dilution 1:100), Cas-3 (Abcam, Cambridge, UK, ab32351; dilution 1:200), and HSP70 (Abcam, Cambridge, UK, ab79852; dilution 1:200).

After primary incubation, slides were treated with a biotinylated secondary antibody (Thermo Fisher Scientific, Waltham, MA, USA, TP-125-BN) and streptavidin–peroxidase complex (Thermo Fisher Scientific, Waltham, MA, USA, TS-125-HR). Visualization was achieved with 3,3′-diaminobenzidine (DAB) (Thermo Fisher Scientific, Waltham, MA, USA, TA-125-HD), followed by rinsing in distilled water. Counterstaining was performed with Harris hematoxylin, and sections were mounted with Entellan.

All stained sections were examined under a light microscope, and semi-quantitative immunostaining scores were assigned as shown in Table S1. Group differences in staining intensity were statistically analyzed as described previously by Ilhan et al. [22].

### 2.7. Reverse Transcription-Polymerase Chain Reaction (qRT-PCR)

RNA was isolated from homogenized tissues using the GeneAll RiboEx™ RNA Isolation Kit (GeneAll Biotechnology, Seoul, Republic of Korea) according to the manufacturer’s protocol. The amount and purity of the RNAs obtained were measured with the BioSpec-nano nanodrop (Shimadzu Ltd., Kyoto, Japan) device. One microgram (1 µg) of RNA was used for cDNA synthesis. cDNA synthesis was performed using the A.B.T.™ cDNA Synthesis Kit (Atlas Biotechnology, Kocaeli, Turkey) in a thermal cycler according to the manufacturer’s protocol. Primer designs were made by detecting specific mRNA sequences and testing possible primer sequences using the NCBI website. The sequences of the primer sequences used are shown in Table S2. Expression levels of genes were measured in a Biorad CFX96 (Hercules, CA, USA) real-time PCR instrument using 2× SYBR green master mix (Nepenthe, Kocaeli, Turkey). In the study, the GAPDH gene was used as a housekeeping gene. The reaction mixture was prepared according to the manufacturer’s protocol to a final volume of 20 µL. The resulting reaction mixture was placed in a qRT-PCR device determined according to the kit manufacturer’s protocol, and each sample was studied in 3 replications. PCR conditions were as follows: initial denaturation at 94 °C for 10 min (1 cycle), followed by 40 cycles of denaturation at 95 °C for 15 s and annealing/extension at 57 °C for 30 s. Relative mRNA levels were calculated by applying the 2^-ΔΔCt^ formula to the normalized results.

### 2.8. Biochemical Examination

Blood samples collected from the rats were placed into gel-containing tubes and centrifuged to separate the serum. The obtained serum samples were stored at −20 °C until biochemical analyses were conducted. Serum creatinine, urea and blood urea nitrogen (BUN) levels were determined spectrophotometrically using a Beckman Coulter AU 5800 automated analyzer (Beckman Coulter, Brea, CA, USA).

### 2.9. Statistical Analysis

All data are presented as mean ± standard deviation. Normality was verified using the Shapiro–Wilk test. Differences among groups were analyzed using one-way ANOVA followed by Tukey’s post hoc test (GraphPad Prism 9.0, San Diego, CA, USA). Statistical significance was accepted at *p* < 0.05. In the figures, * *p* < 0.05, ** *p* < 0.01 and *** *p* < 0.001 denote differences between groups.

## 3. Results

### 3.1. Histopathological Findings

Histopathological assessment of kidney tissues revealed that LPS administration induced severe renal injury, manifested as atrophic glomeruli and renal corpuscles, increased collagen deposition, tubular dilatation, and tubular epithelial degeneration. These pathological changes were significantly more severe in the LPS group compared to controls (for all *p* < 0.001).

Importantly, OMG-3 supplementation (LPS + OMG-3 group) significantly ameliorated all histopathological alterations. Compared to the LPS group, OMG-3 co-treatment markedly reduced the severity of atrophic glomeruli (*p* = 0.007), collagen accumulation (*p* < 0.001), tubular dilatation (*p* < 0.001), and tubular epithelial degeneration (*p* = 0.002).

**Figure 2:**
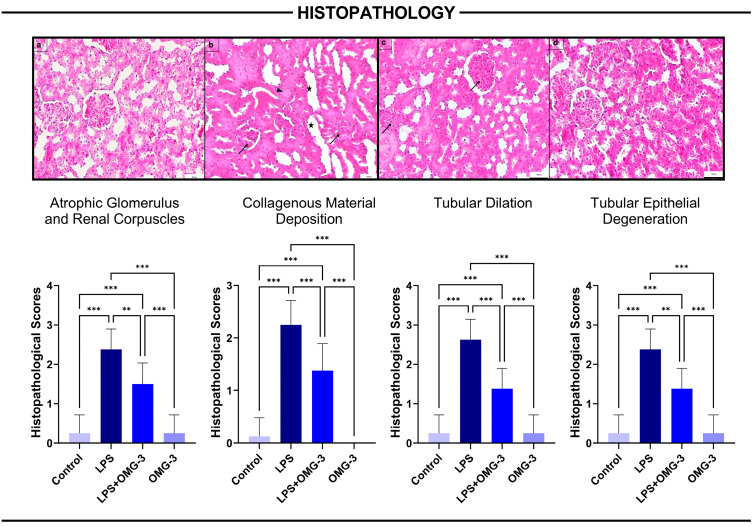
Histopathological evaluation of renal tissues. Representative H&E-stained kidney sections are shown for (**a**) control, (**b**) LPS, (**c**) LPS + OMG-3, and (**d**) OMG-3 groups (scale bar = 100 μm). Severe renal damage was observed in the LPS group, characterized by marked atrophy of glomeruli (arrow), extensive collagen deposition (arrowhead), tubular dilatation (star), and tubular epithelial degeneration. In contrast, co-treatment with OMG-3 (LPS + OMG-3) markedly attenuated these alterations, with histological features approaching those of the control and OMG-3 groups. Data are expressed as mean ± SD (n = 8 per group). One-way ANOVA followed by Tukey’s multiple comparison test was applied (*** = *p* < 0.001, ** = *p* < 0.01). HE: Hematoxylin–eosin, SD: Standard deviation; LPS: Lipopolysaccharide, OMG-3: Omega-3.

No statistically significant differences were observed between the control and OMG-3 groups for any parameter, indicating that OMG-3 alone did not alter baseline renal architecture. These findings collectively demonstrate that OMG-3 exerts a strong protective effect against LPS-induced renal injury, thereby preserving glomerular and tubular integrity (Figure 2).

### 3.2. HSP70 Immunoexpression

Immunohistochemical analysis revealed that LPS administration significantly upregulated *HSP70* expression in renal tissues compared to the control group (*p* < 0.001). The elevated expression was primarily localized to glomerular tufts and tubular epithelial cells, consistent with cellular stress responses.

OMG-3 treatment (LPS + OMG-3) markedly suppressed *HSP70* expression compared to the LPS group (*p* < 0.001), indicating a protective effect against LPS-induced cellular stress. Notably, the *HSP70* levels in the LPS + OMG-3 group remained slightly elevated compared to controls but were significantly lower than those in the LPS group (*p* < 0.001 vs. control).

In the OMG-3 alone group, *HSP70* expression did not differ significantly from the control group (*p* = 0.765), confirming that OMG-3 supplementation by alone does not alter basal *HSP70* expression under physiological conditions.

**Figure 3:**
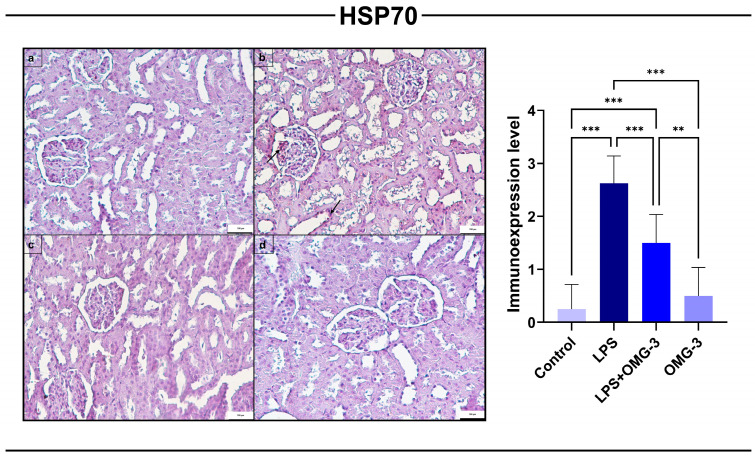
Immunohistochemical expression of *HSP70* in renal tissues. Representative immunohistochemical staining of *HSP70* in (**a**) control, (**b**) LPS, (**c**) LPS + OMG-3, and (**d**) OMG-3 groups (scale bar = 100 μm). Minimal *HSP70* expression was observed in the control and OMG-3 groups. In contrast, the LPS group exhibited markedly increased *HSP70* immunopositivity in glomerular and tubular epithelial cells (arrows). Co-treatment with OMG-3 (LPS + OMG-3) significantly reduced *HSP70* immunoexpression, approaching near-control levels. Data are expressed as mean ± SD (n = 8 per group). One-way ANOVA followed by Tukey’s multiple comparison test was applied (*** = *p* < 0.001, ** = *p* < 0.01). SD: Standard deviation, LPS: Lipopolysaccharide, OMG-3: Omega-3.

Collectively, these findings demonstrate that OMG-3 effectively attenuates LPS-induced *HSP70* overexpression, thereby limiting renal cellular stress responses (Figure 3).

### 3.3. TNF-α Immunoexpression

Immunohistochemical evaluation demonstrated that *TNF-α* expression was significantly elevated in the LPS group compared to controls (*p* < 0.001). Staining was predominantly localized in renal tubular epithelial cells and glomerular compartments, reflecting an enhanced pro-inflammatory response induced by endotoxemia.

OMG-3 supplementation significantly reduced *TNF-α* levels (*p* < 0.001 vs. LPS), indicating a potent anti-inflammatory effect. Although *TNF-α* expression in the LPS + OMG-3 group remained higher than controls (*p* < 0.001), it was significantly lower than in the LPS group, confirming the protective role of OMG-3 against LPS-induced renal inflammation.

In the OMG-3 group, *TNF-α* expression did not differ significantly from that of the control group (*p* = 0.960), indicating that OMG-3 does not affect basal inflammatory status under normal physiological conditions.

**Figure 4:**
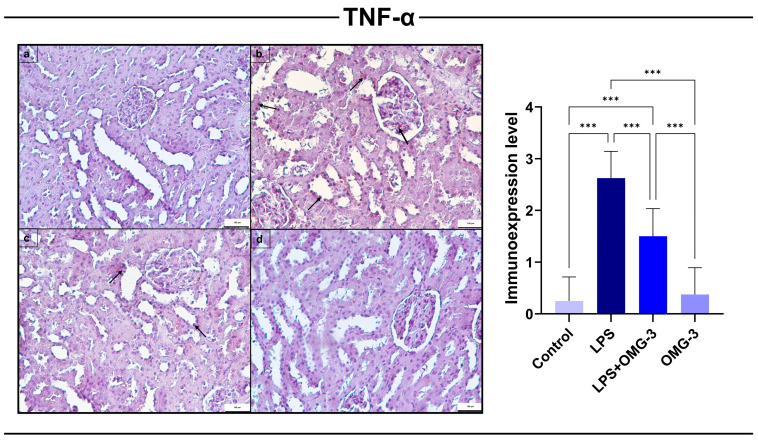
Immunohistochemical expression of *TNF-α* in renal tissues. Representative immunohistochemical staining of TNF-α in (**a**) control, (**b**) LPS, (**c**) LPS + OMG-3, and (**d**) OMG-3 groups (scale bar = 100 μm). Control and OMG-3 groups exhibited negative or minimal *TNF-α* expression. In contrast, LPS exposure caused a strong upregulation of TNF-α immunopositivity, particularly in tubular epithelial cells and glomerular structures (arrows). Co-treatment with OMG-3 (LPS + OMG-3) markedly reduced *TNF-α* expression compared to LPS. Quantitative scoring confirmed that LPS significantly increased *TNF-α* expression relative to the control (*p* < 0.001), while OMG-3 supplementation significantly attenuated this elevation (*p* < 0.001). No significant difference was found between the control and OMG-3 groups (*p* = 0.960). Data are expressed as mean ± SD (n = 8 per group). One-way ANOVA followed by Tukey’s multiple comparison test was applied (*** = *p* < 0.001). SD: Standard deviation, LPS: Lipopolysaccharide, OMG-3: Omega-3, TNF-α: Tumor necrosis factor alpha.

Taken together, these results show that OMG-3 effectively ameliorates LPS-induced upregulation of TNF-α, thereby suppressing renal inflammatory responses (Figure 4).

### 3.4. Cas-3 Immunoexpression

Immunohistochemical analysis demonstrated that LPS administration significantly upregulated Cas-3 expression in renal tissues compared to the control group (*p* < 0.001). This upregulation was localized predominantly to renal tubular epithelial cells, consistent with increased apoptotic activity triggered by endotoxemia.

OMG-3 supplementation (LPS + OMG-3) markedly reduced Cas-3 expression relative to the LPS group (*p* < 0.001). While expression levels in the LPS + OMG-3 group were higher than controls (*p* < 0.001), they were significantly attenuated compared to LPS alone, confirming the protective role of OMG-3.

**Figure 5:**
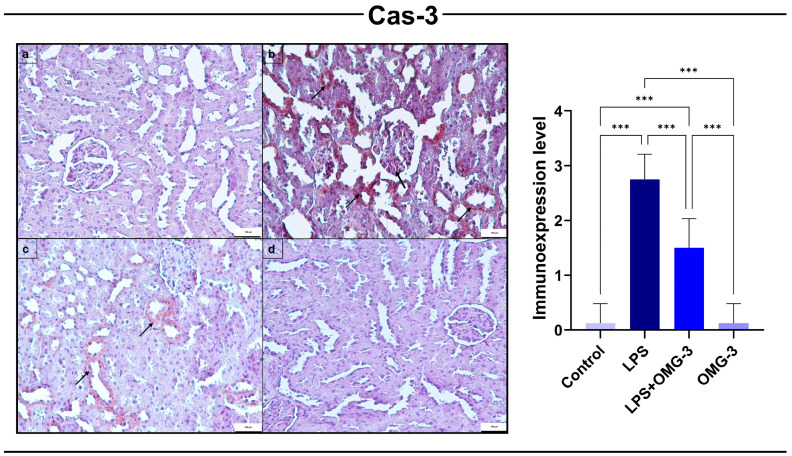
Immunohistochemical expression of *Cas-3* in renal tissues. Representative immunohistochemical staining of Cas-3 in (**a**) control, (**b**) LPS, (**c**) LPS + OMG-3, and (**d**) OMG-3 groups (scale bar = 100 μm). Minimal Cas-3 immunoexpression was observed in the control and OMG-3 groups, indicating negligible apoptotic activity. In contrast, the LPS group exhibited strong Cas-3 positivity, particularly in tubular epithelial cells and glomerular regions (arrows), reflecting enhanced apoptosis. Co-treatment with OMG-3 (LPS + OMG-3) markedly reduced *Cas-3* expression compared to the LPS group. Data are expressed as mean ± SD (n = 8 per group). One-way ANOVA followed by Tukey’s multiple comparison test was applied (*** = *p* < 0.001). SD: Standard deviation, LPS: Lipopolysaccharide, OMG-3: Omega-3.

In the OMG-3 alone group, *Cas-3* levels were nearly identical to those in the control group (*p* > 0.9), indicating that OMG-3 supplementation did not induce apoptosis under physiological conditions (Figure 5).

### 3.5. Oxidative Stress-Related Gene Expressions

Gene expression analyses demonstrated that *LPS* administration markedly suppressed the transcription of *AMPK*, *SIRT1*, *PGC-1α*, *NRF2*, and *FOXO3* genes in renal tissues compared with healthy controls (*p* < 0.01 for SIRT-1, PPGC-1α and NRF2; *p* < 0.001 for *FOXO3* and *AMPK*), confirming a substantial downregulation of the mitochondrial and antioxidant defense pathways. This suppression was effectively reversed by OMG-3 supplementation.

Specifically, FOXO3 and AMPK mRNA levels exhibited the most prominent reductions in the LPS group, while treatment with OMG-3 significantly restored these expressions toward baseline (*p* = 0.033 for FOXO3 and *p* = 0.006 vs. LPS). *PGC-1α* expression, a key regulator of mitochondrial biogenesis, was also decreased in LPS-treated rats (*p* = 0.002 vs. control) and improved in both the LPS + OMG-3 and OMG-3 only groups (respectively; *p* = 0.05 and *p* = 0.055 vs. LPS).

**Figure 6:**
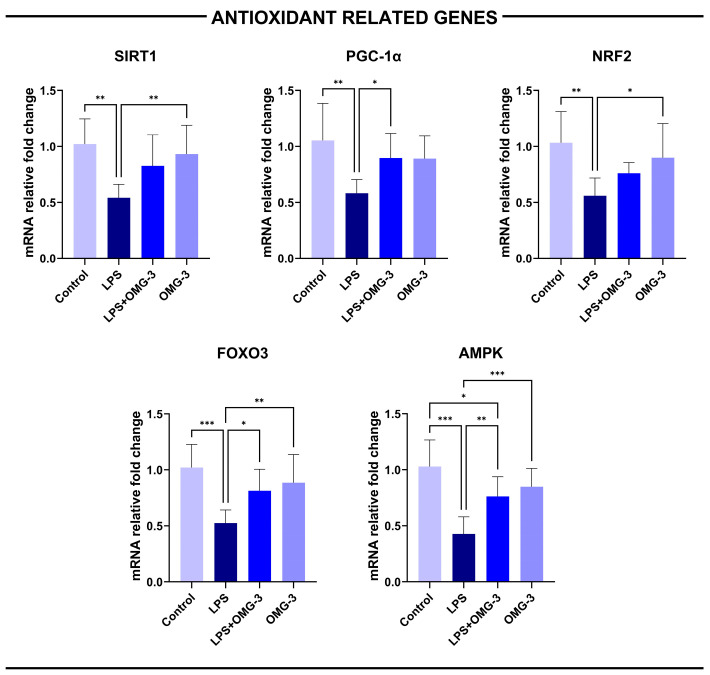
Relative mRNA expression of *AMPK*, *SIRT1*, *PGC-1α*, *NRF2*, and *FOXO3* genes in renal tissue following LPS challenge and OMG-3 treatment. Bar graphs illustrate the gene expression profiles of key oxidative stress-responsive transcriptional regulators across experimental groups. LPS exposure markedly downregulated *SIRT-1*, *PGC-1α*, *NRF2*, *FOXO3*, and *AMPK* compared with the control group, reflecting impaired antioxidant defense and mitochondrial regulatory signaling. Co-treatment with OMG-3 (LPS + OMG-3) significantly restored the expression of all examined genes, approaching or surpassing baseline levels, while the OMG-3–alone group exhibited preserved or mildly elevated expression, confirming its intrinsic cytoprotective potential. Data are presented as mean ± SD. One-way ANOVA followed by Tukey’s post hoc test was used for statistical comparisons (* *p* < 0.05, ** *p* < 0.01, *** *p* < 0.001). SD: Standard deviation; LPS: Lipopolysaccharide; OMG-3: Omega-3.

*AMPK* expression levels in the LPS + OMG-3 group were higher than those in the LPS group (*p* = 0.035), yet remained lower than in controls, indicating that OMG-3 partially restored *AMPK* expression suppressed by LPS (Figure 6).

### 3.6. Serum Analysis

**Figure 7:**
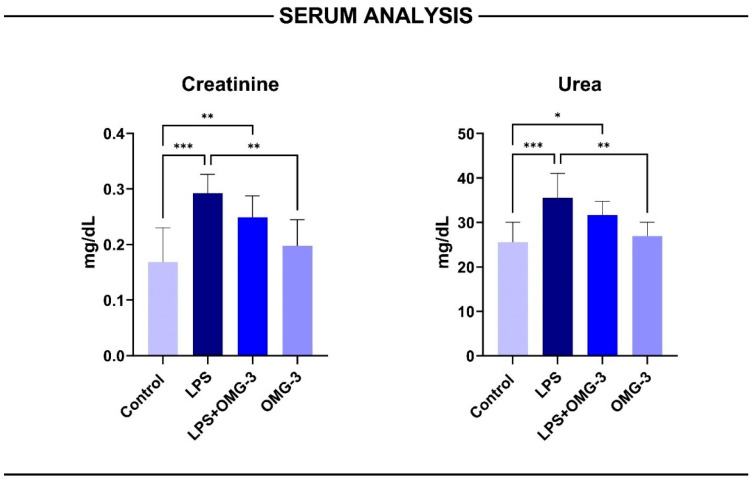
Serum biochemical parameters in LPS-induced acute kidney injury and the effect of OMG-3 treatment. Bar graphs illustrate serum creatinine (**left**) and urea (**right**) concentrations (mg/dL) in control, LPS, LPS + OMG-3, and OMG-3 groups. LPS administration significantly elevated both creatinine and urea levels compared with the control group. OMG-3 co-treatment (LPS + OMG-3) markedly reduced these elevations, restoring biochemical parameters toward control values. No significant differences were observed between the control and OMG-3 groups (ns > 0.05), indicating that OMG-3 alone did not alter baseline renal function. Data are presented as mean ± SD (n = 8 per group). One-way ANOVA followed by Tukey’s post hoc test was used for statistical comparisons (* *p* < 0.05, ** *p* < 0.01, *** *p* < 0.001). SD: Standard deviation; LPS: Lipopolysaccharide; OMG-3: Omega-3.

Serum biochemical analyses demonstrated that LPS administration markedly impaired renal function, as evidenced by increased creatinine and urea concentrations compared to the control group (*p* < 0.001 for both). OMG-3 co-treatment significantly attenuated these elevations, restoring serum indices toward baseline (*p* < 0.01 vs. LPS). No significant differences were observed between the control and OMG-3 groups (ns > 0.05), indicating that OMG-3 supplementation alone did not affect normal renal physiology (Figure 7).

Despite the biochemical recovery observed in the LPS + OMG-3 group, the LPS-treated rats displayed variable serum responses that were not always proportional to histopathological injury. This paradoxical pattern where creatinine and urea levels appear lower or partially normalized despite evident tissue damage may reflect early subacute recovery or transient hyperfiltration associated with LPS-induced hemodynamic alterations.

Collectively, these results confirm that OMG-3 supplementation exerts a nephroprotective effect by mitigating LPS-induced functional deterioration and normalizing serum markers of renal performance.

## 4. Discussion

The present study demonstrated that OMG-3 fatty acid supplementation exerts robust renoprotective, anti-inflammatory, and antioxidant effects in an LPS-induced AKI model, as evidenced by histopathological, immunohistochemical, and gene expression findings. These results highlight the pivotal role of the AMPK/SIRT1/PGC-1α/FOXO3/HSP70 and NRF2 axis in mediating OMG-3-driven mitochondrial protection and attenuation of NF-κB-dependent inflammatory cascades [16,23].

LPS exposure induced severe renal parenchymal injury characterized by glomerular atrophy, tubular epithelial degeneration, tubular dilatation, collagenous material accumulation, and interstitial inflammation findings consistent with classical sepsis-associated nephropathy [3,24]. Such morphological deterioration reflects microvascular dysfunction, oxidative stress, and apoptotic loss of tubular epithelial cells, which collectively impair glomerular filtration and tubular reabsorption [21].

OMG-3 treatment significantly reversed these histological lesions, indicating preservation of glomerular architecture and tubular integrity. This structural improvement can be attributed to OMG-3’s membrane-stabilizing and anti-fibrotic properties, previously reported to inhibit extracellular matrix accumulation and reduce transforming growth factor beta (TGF- β) activity [25]. The attenuation of collagen deposition observed in the current study agrees with evidence that EPA and DHA reduce collagen I/III accumulation by inhibiting fibroblast activation and dampening pro-inflammatory signaling such as NF-κB and TGF-β-mediated pathways [26].

Immunohistochemical analysis revealed that LPS markedly increased *TNF-α* expression in glomerular and tubular epithelial cells, reflecting activation of the innate immune response and NF-κB signaling [27]. *TNF-α* is a principal cytokine mediating endothelial dysfunction and leukocyte infiltration in septic AKI [28]. In contrast, OMG-3 supplementation substantially reduced TNF-α immunoreactivity, consistent with its known ability to inhibit NF-κB nuclear translocation and inhibitor kappa B alpha degradation [28,29]. Mechanistically, this anti-inflammatory effect may involve SIRT1 activation, which deacetylates the p65 subunit of NF-κB, thereby suppressing transcription of pro-inflammatory genes [30].

The transcriptional upregulation of SIRT1 observed in this study strongly supports this mechanism. Enhanced SIRT1 activity, coupled with increased AMPK phosphorylation, creates a negative feedback loop that limits TNF-α production and cytokine storm progression [31]. Thus, OMG-3’s anti-inflammatory protection appears tightly linked to restoration of the AMPK/SIRT1 axis, which modulates NF-κB-driven inflammatory gene networks.

Unlike conventional NF-κB inhibitors that primarily target downstream inflammatory signaling pathways, OMG-3 fatty acids demonstrate a unique dual mechanism of action. Our findings suggest that beyond providing mere anti-inflammatory suppression, OMG-3 simultaneously actively restores mitochondrial biogenesis and antioxidant defense through the transcriptional upregulation of the AMPK/SIRT1/PGC-1α axis. This multi-targeted approach—coupling immune regulation with metabolic reprogramming—distinguishes OMG-3 from single-target inhibitors and underscores its novelty as a comprehensive cytoprotective agent capable of restoring cellular bioenergetics alongside inflammation control.

*Cas-3* immunopositivity was significantly elevated following LPS administration, signifying extensive apoptotic activity within tubular cells. Excessive activation of executioner caspases, such as *Cas-3*, has been associated with mitochondrial dysfunction and cytochrome c release during septic renal injury [32]. OMG-3 supplementation effectively reduced Cas-3 expression, implying strong anti-apoptotic potential.

This effect likely results from the restoration of the SIRT1/FOXO3 signaling axis. FOXO3 is a transcription factor that promotes cellular resistance to oxidative stress by upregulating antioxidant enzymes [4]. However, when deacetylation by SIRT1 is suppressed under oxidative stress, FOXO3 becomes transcriptionally inactive, leading to apoptotic vulnerability [33]. The upregulation of both SIRT1 and FOXO3 mRNA levels in the OMG-3-treated group suggests reactivation of FOXO3-mediated survival signaling, consistent with reduced Cas-3 expression and improved tubular morphology. Similar renoprotective mechanisms have been described where OMG-3 or SIRT1 agonists enhance FOXO3 transcription to suppress Cas-3 and BAX (BCL2-Associated X) and Apoptosis Regulator-mediated apoptosis [16,23].

LPS challenge caused a pronounced increase in HSP70 immunoreactivity, reflecting cellular stress and protein denaturation in renal epithelial cells [7,33]. HSP70 upregulation represents a compensatory defense against oxidative and inflammatory injury but also serves as an indicator of mitochondrial unfolded protein response activation [34]. OMG-3 supplementation significantly reduced *HSP70* expression, suggesting attenuation of oxidative stress and restoration of protein-folding homeostasis. This finding parallel reports that OMG-3 enhances mitochondrial chaperone activity by improving NAD^+^-dependent metabolic signaling through SIRT1 and AMPK [6,16]. The normalization of *HSP70* levels therefore reflects reduced mitochondrial burden and improved proteostasis.

The gene expression analysis provided direct molecular insight into the mechanisms underlying OMG-3’s renoprotective effects. LPS administration caused significant downregulation of AMPK, SIRT1, PGC-1α, NRF2, and FOXO3, demonstrating disruption of the mitochondrial biogenesis-antioxidant axis. These results align with previous findings showing that sepsis suppresses AMPK activity and SIRT1 deacetylation, leading to impaired mitochondrial metabolism and ROS accumulation [35,36].

OMG-3 treatment effectively reversed this downregulation, restoring gene expression toward baseline. The observed increases in AMPK and SIRT1 transcript levels indicate transcriptional recovery and NAD^+^ turnover improvement, both essential for mitochondrial maintenance [16,22]. SIRT1-mediated deacetylation of PGC-1α promotes transcription of mitochondrial DNA replication factors, while PPGC-1α coactivates NRF2 to upregulate antioxidant enzymes such as HO-1, SOD, and GPX4 [37]. Thus, the synchronized elevation of PGPGC-1α, NRF2, and FOXO3 in the OMG-3-treated group suggests a coordinated transcriptional response related to mitochondrial resilience and antioxidant defense.

Consistent with these molecular alterations, serum biochemical indices corroborated the renoprotective role of OMG-3. The marked elevations of creatinine, urea, and BUN following LPS exposure are hallmarks of impaired glomerular filtration and tubular dysfunction, aligning with the observed histopathological damage [27,38]. Notably, OMG-3 treatment normalized these parameters, suggesting restored renal perfusion and reabsorption capacity. This biochemical improvement likely stems from the attenuation of oxidative and inflammatory insults within the renal microenvironment, further substantiating the structural and molecular protection afforded by OMG-3.

The molecular and histopathological findings of this study are mechanistically coherent. The decrease in *TNF-α* and *Cas-3* expression parallels the upregulation of SIRT1 and FOXO3, confirming that anti-inflammatory and anti-apoptotic effects share a common signaling foundation. Similarly, the reduction in *HSP70* immunoreactivity correlates with restoration of *AMPK* and *NRF2* expression, consistent with decreased oxidative load and improved mitochondrial function. Collectively, these cross-level correlations suggest that OMG-3 exerts multi-target protection through the transcriptional upregulation of *AMPK*, *SIRT1*, *PGC-1α*, *NRF2* and *FOXO3* reactivation, which mitigates inflammatory (TNF-α), apoptotic (Cas-3), and stress (HSP70) responses at both transcriptional and tissue levels.

Several recent studies corroborate these observations. In a renal fibrosis model, Han et al. demonstrated that OMG-3 polyunsaturated fatty acids activated the AMPK/SIRT1 signaling pathway, thereby attenuating oxidative stress and extracellular matrix accumulation in kidney tissue [39]. Similarly, in a nephrectomized rat model, Son et al. reported that *OMG-3* supplementation upregulated SIRT1/3 and PGC-1α, improving mitochondrial biogenesis and redox balance [16]. Collectively, these findings reinforce the current data, suggesting that OMG-3-induced metabolic reprogramming and antioxidant defense are conserved mechanisms across organ systems subjected to inflammatory or endotoxin stress.

Acute kidney injury (AKI) represents one of the most critical determinants of adverse outcomes in sepsis, with affected individuals exhibiting markedly higher mortality rates compared with septic patients without AKI [40]. Considering this clinical burden, therapeutic strategies capable of mitigating organ dysfunction are of great importance. In this context, the ability of OMG-3 fatty acids to modulate mitochondrial bioenergetics, reduce oxidative stress, and regulate inflammatory signaling pathways suggests that they may complement current standard-of-care interventions and hold significant potential to improve renal outcomes in patients with sepsis.

## 5. Study Limitations

Although the present study provides comprehensive histopathological, immunohistochemical, and molecular evidence supporting the renoprotective effects of OMG-3 fatty acids against LPS-induced injury, several limitations should be acknowledged. First, the experimental design focused primarily on acute-phase responses (72 h post-LPS); therefore, the long-term outcomes of OMG-3 supplementation on renal repair, fibrosis progression, or functional recovery were not assessed.

Second, while the study explored major mitochondrial and antioxidant regulators (AMPK, SIRT1, PGC-1α, NRF2, and FOXO3), protein-level confirmation through Western blotting or enzyme-linked immunosorbent assay was not performed due to equipment limitations. This may have limited direct validation of transcriptional findings at the post-translational level.

Additionally, while HSP70 protein expression was evaluated via immunohistochemistry as a key stress marker, its gene expression levels were not analyzed by qRT-PCR. This creates a gap in the multi-level analysis of this specific marker compared to the other pathway components.

Third, the study utilized only male Wistar rats, which precludes assessment of potential sex-related differences in inflammatory or metabolic responses to OMG-3. Considering that estrogen and androgen signaling influence mitochondrial function and NF-κB activity, future studies should include both sexes to enhance translational value.

Finally, the study employed a single dose and duration of OMG-3 administration. Although this regimen effectively mitigated acute renal injury, dose–response and chronic supplementation studies could further optimize therapeutic translation. Furthermore, a vehicle-only control group (e.g., corn oil without OMG-3) was not included. Although the volume of corn oil used as a carrier was minimal, its potential minor contribution to the observed effects cannot be completely ruled out.

Despite these limitations, the integration of multi-level analyses—histopathology, immunohistochemistry, and gene expression—provides robust mechanistic evidence that OMG-3 modulates the AMPK, SIRT1, PGC-1α, NRF2 and FOXO3 signaling axis to protect renal tissue against sepsis-associated oxidative and apoptotic damage.

Another limitation is the absence of mitochondrial functional assays (ATP production, membrane potential, or oxygen consumption), which would further strengthen the mechanistic link between OMG-3 and mitochondrial restoration. Additionally, Western blot validation of AMPK, SIRT1, PGC-1α, NRF2, and FOXO3 proteins would provide confirmatory evidence supporting the transcript-level changes.

## 6. Conclusions

In summary, OMG-3 fatty acid supplementation markedly alleviated LPS-induced renal injury by suppressing inflammatory (TNF-α) and apoptotic (Cas-3) pathways and restoring the transcriptional levels of mitochondrial biogenesis and antioxidant defense genes, including AMPK, SIRT1, PGC-1α, NRF2 and FOXO3 signaling. The consistency between morphological, immunohistochemical, and genetic findings supports the hypothesis that OMG-3 protects renal tissue by restoring mitochondrial redox equilibrium and inhibiting NF-κB-driven cytokine responses. These results highlight OMG-3 as a promising adjuvant strategy for preventing and treating sepsis-associated AKI. Collectively, these results position OMG-3 as a promising adjuvant strategy for preventing early mitochondrial failure and inflammation-driven tissue injury in sepsis-associated AKI.

## Data Availability

The data that support the findings of this study are available from the corresponding author upon reasonable request.

## Supplementary Materials

The following supporting information can be downloaded at: https://www.mdpi.com/article/10.3390/nu18040618/s1, Table S1: Histopathological and immunohistochemical scores of kidney tissues. Table S2: Primer sequences, product size and accession numbers of genes.

## Abbreviations

AKI	Acute kidney injury
AMPK	Adenosine monophosphate-activated protein kinase
Cas-3	Caspase 3
DHA	Docosahexaenoic acid
DAB	3,3′-diaminobenzidine
EPA	Eicosapentaenoic acid
FOXO3	Forkhead box O3
GPX4	Glutathione peroxidase 4
HSP70	Heat shock protein 70
HO-1	Heme oxygenase 1
IL-1β	Interleukin 1 beta
IL-6	Interleukin 6
LPS	Lipopolysaccharide
NF-κB	Nuclear factor kappa beta
NRF2	Nuclear factor erythroid 2-related factor 2
OMG-3	Omega-3
PBS	Phosphate-buffered saline
PGC-1α	Peroxisome proliferator-activated receptor gamma coactivator-1 alpha
qRT-PCR	Quantitative real-time polymerase chain reaction
ROS	Reactive oxygen species
SIRT1	Sirtuin 1
SOD	Superoxide dismutase
TGF-β	Transforming growth factor beta
TLR4	Toll-like receptor 4
TNF-α	Tumor necrosis factor alpha
