# Supplementary material for: Omega-3 Fatty Acids Attenuate LPS-Induced Acute Kidney Injury via Activation of AMPK/SIRT1/PGC-1α/NRF2/FOXO3 Signaling and Suppression of NF-κB-Mediated Inflammation
Source: Nutrients. 2026 Feb 13;18(4):618. doi: 10.3390/nu18040618 (PMC12943360; doi:10.3390/nu18040618)
Supplement: Supplementary file 1 [file nutrients-18-00618-s001.zip › nutrients-4118651-supplementary.pdf]

Table S1. *Histopathological and immunohistochemical scores of kidney tissues.*

	Immunohistochemical Evaluations	Histopathological Evaluations
0 / –	Negative staining,	None
1 / +	Focal and weak staining,	Mild
2 / ++	Diffuse and moderate staining,	Moderate
3 / +++	Intense and strong staining.	Severe

Table S2. *Primer sequences, product size and accession numbers of genes.*

Genes	Primer sequence	Product size	Accession number
GAPDH (HouseKeeping)	F: AGTGCCAGCCTCGTCTCATA	248 bp	NM_017008.4
GAPDH (HouseKeeping)	R: GATGGTGATGGGTTTCCCGT	248 bp	NM_017008.4
AMPK	F: TCGGCAAAGTGAAGATTGGAG	308 bp	NM_023991.2
AMPK	R: GTAGTCCACGGCAGACAGAA	308 bp	NM_023991.2
SIRT1	F: GGTAGTTCCTCGGTGTCCT	152 bp	NM_001414959.1
SIRT1	R: ACCCAATAACAATGAGGAGGTC	152 bp	NM_001414959.1
PGC- 1α	F: CGCACAACTCAGCAAGTCCTC	263 bp	XM_039092494.1
PGC- 1α	R: CCTTGCTGGCCTCCAAAGTCTC	263 bp	XM_039092494.1
FOXO3	F: GGGAGGAGGAGGAATGTGGA	164 bp	NM_001106395.1
FOXO3	R: CTACCTCGGCTCCTTCCCT	164 bp	NM_001106395.1
NRF2	F: GCCTTCCTCTGCTGCCATTAGTC	126 bp	NM_001399173.1
NRF2		126 bp	NM_001399173.1

	R: TCATTGAACTCCACCGTGCCTTC		
